# Disease-specific differences in the use of traditional Korean medicine in Korea

**DOI:** 10.1186/s12906-015-0657-9

**Published:** 2015-05-03

**Authors:** In-Hwan Oh, Seok-Jun Yoon, Minjung Park, SoHee An

**Affiliations:** Department of Preventive Medicine, School of Medicine, Kyung Hee University, 1, Seoul, Heogi-Dong, Dongdaemun-gu 130-701 Korea; Department of Preventive Medicine, College of Medicine, Korea University, 5-Ga, Anam-dong, Seoul, Seongbuk-gu 136-705 Korea; Graduate School of Public Health, Seoul National University, Seoul, Daehak-dong, Gwanak-gu 151-742 Korea

**Keywords:** CAM, Traditional Korean medicine, Preferences, Cost

## Abstract

**Background:**

Though traditional Korean medicine plays an important role in the Korean parallel health care system, there is limited information about the preference and usage of traditional Korean medicine compared to Western medicine because they have different disease classification systems. The aim of this study is to determine the relative preference for traditional Korean medicine using data acquired nationwide.

**Methods:**

Data from the 2008 Korea Health Panel were analyzed to determine the preference of medical services by disease. The use of traditional Korean medicine use is defined by the type of medical institution they used. Disease types, number of visits and out of pocket expenditures were analyzed.

**Results:**

Traditional Korean medicine was used in only a small number of cases that were emergencies or hospitalization. However, in terms of outpatient services, traditional Korean medicine was used in 7.8% of all cases and represented 9.9% of total medical costs. Among disease groups, traditional Korean medicine use was higher in patients with nervous system and musculoskeletal system diseases. And patients with musculoskeletal and nervous system diseases such as arthrosis were the most likely to use traditional Korean medicine particularly in an outpatient setting.

**Conclusions:**

Korean characteristics of service use resemble the complementary and alternative medicine use in other countries in terms of disease group, and the complementary and alternative medicine should be considered to estimate the burden of disease in countries with parallel health care systems, such as Korea. This is the first study determined the actual preference of traditional Korean medicine for specific chronic diseases.

## Background

The use of complementary and alternative medicine (CAM) is a worldwide phenomenon [1]. In some Asian and African countries, 80% of the population use CAM for primary health care. Even in many developed countries, 70–80% of the population use at least some form of CAM [1]. For example, in the United States, approximately 38% of adults and 12% of children used some form of CAM in 2007 [2]. In Europe, approximately 35% of cancer patients reported the use of some form of CAM, though prevalence of use varies by country [3].

CAM could be defined as “a group of diverse medical and health interventions, practices, or disciplines that are not generally considered part of conventional medicine” [4]. It could be grouped as natural products or mind and body practices. Also traditional medicine such as Ayurvedic medicine, traditional Chinese medicine was included in CAM [1]. Korea has its own traditional medicine like Japan, Malaysia and Vietnam [5]. Though traditional Korean medicine (TKM) has some similarities to traditional Chinese medicine, Koreans have developed their own methodology for treating diseases, such as Sasang constitutional medicine [6]. TKM is primarily composed of acupuncture, moxibustion and herbal medicine [6]. Korea has a parallel health care system consisting of TKM and Western medicine [7,8]. The general system of TKM resembles Western medicine. They each have their own six-year undergraduate education and subsequent licensing examination. There are TKM hospitals and clinics in much the same way there are Western hospitals and clinics. In 2008, the numbers of Western medicine doctors and TKM doctors were 95,013 and 17,473 respectively. Also the number of Western medicine hospital including a tertiary and general hospital was 2,195. On the contrary, the number of TKM hospital was only 148 [9,10]. Services such as acupuncture that are provided by TKM doctors (generally referred to as doctors of Korean medicine (DKM)) are partially covered by the National Health Insurance of Korea. In a national survey of Korean citizens, the prevalence of CAM use was approximately 29% in 1999. Of those CAM users, approximately half (15%) received medical attention from an DKM [11]. A more recent survey of the Korean population conducted in 2006 indicated that the prevalence of CAM use was 74.8% and the prevalence of TKM use was at 31.6% [12]. The medical cost of TKM, defined as the cost of TKM hospitals and clinics, is approximately one billion dollars, which represents 3.9% of total medical costs covered by national health insurance according to the 2008 national health insurance statistics [10].

Though TKM plays a substantial role in the Korean health care system and the cost of TKM is not negligible, it is difficult to determine the proportion of Korean citizens using TKM to treat a specific disease or condition. Though it is possible to estimate TKM usage in terms of total medical cost, the preference of each patient for TKM versus Western medicine for a given disease is not well understood. Although it is known that the use of TKM is higher for the treatment of musculoskeletal, endocrine, obstetric and gynecological conditions, the prevalence of using TKM to treat specific diseases has not yet been reported [11]. This is largely because there are different disease classification systems for TKM and Western medicine. In Western medicine, diseases are generally classified by international classification of diseases (ICD) codes [13]. In Korea, the Korean version of ICD-10 (KCD) is currently applied when diagnosing diseases using Western medicine, while the diagnosis of diseases using TKM typically involves the TKM diagnosis system. Since TKM has a distinctive, diagnostic approach, Pattern Identification (PI), Disease classification system of TKM is composed to reflect the PI. It categorized the body condition according to the quantity of Qi or Blood such as deficiency of Qi or blood or Sasang constitution such as So-eumin, So-yangin, Tae-umin and Tae-yang-in). Therefore, though the total number of services or total cost of TKM is available, disease-specific data is generally not.

A standard matching algorithm between ICD-codes and the TKM diagnosis system was developed to account for the differences between Western and TKM disease classifications [14]. However, according to this algorithm, one TKM diagnosis code corresponds to multiple ICD codes, making practical use of the algorithm almost impossible [15]. For example, the TKM diagnosis of back pain could be matched to the ICD code for kyphosis and lordosis (M40), other intervertebral disc disorders (M51), coxarthrosis (M16), or a sprain or strain of the lumbar spine (S33.5). The TKM diagnosis system was reconstructed in 2009 to address some of these shortcomings [16]. In the new diagnosis system, a diagnosis should be made based on the KCD in principle even when TKM is used. But specific conditions that cannot be classified using original KCD are classified as U20-98 of KCD. Thus, the TKM diagnosis system is now integrated with ICD codes, and this new classification system is called KCD-6. However, because there is still a special diagnosis code for TKM, patients with the same symptoms may still be classified under different diagnosis codes depending on the institution. Therefore, it is still difficult to determine patient preferences for using TKM or Western medicine to treat a specific disease. In Korea Health Panel, each disease was defined by patients during a survey conducted by trained reviewers as part of the Panel. Disease classification was based on a grouping system that includes 298 diseases. This system is used for statistical purposes for national health insurance claims and is matched with KCD codes [17]. This made it possible for the Korea Health Panel to determine a patient’s preference for medical services by disease. In this study, the preference and relative cost of TKM by specific disease was assessed using data collected by the Korea Health Panel in order to better understand the preference of TKM in the parallel health care system in Korea.

## Methods

In this study, results from the integrated version of the 2008 Korea Health Panel (β-version 1.1.1) were analyzed to determine medical service use by disease [18]. Korea Health Panel is a nationwide survey to catch the health service use behavior of Korean. In integrated version of the 2008 Korea Health Panel, the surveyed were composed of 21,787 of 7,006 families that were selected by two-stage cluster sampling from the Korean census in 2005. Their health service use behaviors such as inpatient and outpatient service use were examined by trained interviewer. A detailed description of the Korea Health Panel was presented elsewhere [18,19]. Due to the parallel system of Korea, TKM is only provided in TKM medical institutions. Some institutions such as health care centers were the exceptions and mixed provision is allowed. Therefore, the use of Western medicine and TKM was classified based on the medical institution where a patient sought treatment. Patients were asked “What medical institution did you use to treat this specific case?” and a trained interviewer recorded the kind of the institution. The use of a tertiary hospital, general hospital, or hospital and clinic was classified as Western medicine use and the use of TKM hospitals and clinics was classified as TKM use. The use of dental hospitals and clinics was defined as dental service use, and the use of other medical institutions not located in Korea, health care centers and sub-centers, health center county hospitals, and long term care hospitals was classified as other service use due to their mixed provision of both Western medicine and TKM.

TKM was frequently used for outpatient services and a disease-specific preference was defined. Patient diagnoses were acquired from survey data, and diagnoses were then classified by the disease classification code of the Korea Health Panel. When patients used a clinic for multiple reasons, the primary diagnosis code was used for classification. The top 20 diseases for which TKM was used were then determined. The disease group specific preference was also analyzed using a 20-disease group system used by the Korea Health Panel [18]. The cost and cases of selected diseases and disease groups were investigated.

The frequency of TKM use by patients with chronic diseases was investigated using data from the Korea Health Panel also. Patients were asked whether they had a chronic disease and if the diseases had been diagnosed by a physician in the panel. Among the determined top 20 diseases for TKM use, acute diseases and injuries by diseases characteristics are excluded. Data describing chronic diseases and outpatient services were matched to patient identifier. The medical uses of patients due to their specific chronic disease were only included in the analysis. As a result, the top ten chronic diseases included arthrosis (M15–M19), soft tissue disorders (M60–M79), other diseases of the skin and subcutaneous tissues (L00–L08), gastritis and duodenitis (K29) and other dorsopathies (M40–M49, M53–54). The numbers of outpatients visits due to their chronic disease of each patient were identified and annual average number and cost of outpatients service use were estimated. Cost was measured by the Korea Health Panel survey which determined costs paid directly by patients, including copayment and the cost of care not covered by national health insurance. Costs paid by the insurer were excluded in the survey. And the cost of herbal medicines at TKM hospitals and clinics was included, but the cost of prescribed medications from a pharmacy was excluded, as was the cost of national health insurance. For top 10 frequent TKM use diseases, the average number and cost of outpatient services was provided by Western medicine, dental and other medical service, TKM respectively. This study used public data from the Korea Health Panel that did not include any personal identification, and the survey conformed to local legislation and the Declaration of Helsinki. The cost, originally measured in Korean won, was converted to US dollars (1 $=1108.9 won) [20]. SAS 9.2 (SAS institute, Inc., Cary, NC) was used for data analysis.

## Results

Of the 2,704 hospitalized cases in 2008, 2,624 (97.0 ± 0.33%) cases were Western medicine use cases. Sixty (2.2 ± 0.28%) were dental and other services use cases, and 20 were (0.7 ± 0.16%) TKM use cases. TKM represented 1.0% of total in-patient medical costs. TKM use for emergency services was similar; only 0.4 ± 0.14% of those surveyed used TKM, representing 0.1% of total emergency medical costs for 2008.

Of the 238,202 cases that used outpatient services in 2008, 204,110 cases were Western medical services use cases, 15,594 were dental services and other services use cases, and 18,498 (7.8 ± 0.05%) were TKM use cases (Table 1). According to the Korea Health Panel survey, 0.4 million US dollars were spent on TKM in 2008, which represents 9.9% of the total costs for outpatient services. Thus, for outpatient services, the proportion of TKM service costs is higher than the proportion of TKM use cases. The cost of Western medical services was 2.1 million US dollars, representing 55.4% of the cost of total outpatient services. The cost of dental and other medical services was 34.7% of the cost of total outpatient services (Table 1).

**Table 1:** **Number of outpatient cases and cost of outpatient medical services by disease group** ^**a)**^

**Disease group (ICD code)** ^**c**^	**Western medicine**				**Traditional Korean medicine**				**Dental and other medical service use**				**Total**	
	**Number**	**Proportion of number (S.E.)**	**Cost** ^**b)**^	**Proportion of cost**	**Number**	**Proportion of number (S.E.)**	**Cost** ^**b)**^	**Proportion of cost**	**Number**	**Proportion of number (S.E.)**	**Cost** ^**b)**^	**Proportion of cost**	**Number**	**Cost** ^**b)**^
Certain infectious and parasitic diseases (A00-B99)	3,094	99.0%	31.6	97.8%	24	0.7%	0.7	2.2%	7	0.2%	0.0	0.1%	3,124	32.3
		(0.17)				(0.15)				(0.08)				
Neoplasms (C00–D48)	3,108	98.6%	163.6	98.3%	30	1.0%	2.6	1.6%	13	0.4%	0.1	0.1%	3,151	166.4
		(0.21)				(0.17)				(0.11)				
Diseases of the blood and blood-forming organs and certain disorders involving the immune system (D50–D89)	353	97.5%	3.5	71.7%	9	2.5%	1.4	28.3%	0	0.0%	0.0	0.0%	362	4.9
		(0.82)				(0.82)				(0.00)				
Endocrine, nutritional and metabolic diseases (E00–E90)	8,204	98.6%	78.9	93.4%	91	1.1%	5.4	6.4%	25	0.3%	0.2	0.2%	8,320	84.5
		(0.13)				(0.11)				(0.06)				
Mental and behavioral disorders (F00–F99)	3,080	94.2%	48.6	89.6%	163	5.0%	5.2	9.6%	26	0.8%	0.5	0.8%	3,269	54.2
		(0.41)				(0.38)				(0.16)				
Diseases of the nervous system (G00–G99)	2,465	74.5%	42.3	72.6%	830	25.1%	15.9	27.3%	15	0.5%	0.1	0.1%	3,310	58.2
		(0.76)				(0.75)				(0.12)				
Diseases of the eye and adnexa (H00–H59)	7,625	99.1%	93.5	98.7%	64	0.8%	1.2	1.3%	5	0.1%	0.0	0.1%	7,694	94.8
		(0.11)				(0.10)				(0.03)				
Diseases of the ear and mastoid process (H60–H95)	2,790	98.1%	19.6	93.4%	52	1.8%	1.4	6.6%	1	0.1%	0.0	0.1%	2,843	21.0
		(0.25)				(0.25)				(0.04)				
Diseases of the circulatory system (I00–I99)	25,543	93.2%	159.4	88.6%	1,696	6.2%	19.5	10.9%	174	0.6%	0.9	0.5%	27,413	179.8
		(0.15)				(0.15)				(0.05)				
Diseases of the respiratory system (J00–J99)	58,716	98.7%	207.9	89.4%	736	1.2%	21.8	9.4%	63	0.1%	2.8	1.2%	59,515	232.5
		(0.05)				(0.05)				(0.01)				
Diseases of the digestive system (K00–K93)	10,301	43.3%	175.2	12.2%	437	1.8%	13.1	0.9%	13,080	54.9%	1,243.0	86.8%	23,818	1,431.3
		(0.32)				(0.09)				(0.32)				
Diseases of the skin and subcutaneous tissues (L00–L99)	7,670	96.1%	72.4	81.6%	305	3.8%	14.9	16.8%	7	0.1%	1.4	1.6%	7,982	88.7
		(0.22)				(0.21)				(0.03)				
Diseases of the musculoskeletal system and connective tissues (M00–M99)	37,460	77.1%	279.6	69.9%	10,909	22.4%	119.6	29.9%	237	0.5%	0.9	0.2%	48,606	400.2
		(0.19)				(0.19)				(0.03)				
Diseases of the genitourinary system (N00–N99)	7,145	91.4%	122.2	88.7%	191	2.4%	11.1	8.0%	485	6.2%	4.5	3.2%	7,821	137.7
		(0.32)				(0.17)				(0.27)				
Pregnancy, childbirth and the puerperium (O00–O99)	199	94.8%	8.0	96.0%	11	5.2%	0.3	4.0%	0	0.0%	0.0	0.0%	210	8.3
		(1.54)				(1.54)				(0.00)				
Certain conditions originating in the perinatal period (P00–P96)	54	100.0%	1.8	100.0%	0	0.0%	0.0	0.0%	0	0.0%	0.0	0.0%	54	1.8
		(0.00)				(0.00)				(0.00)				
Congenital malformations, deformations and chromosomal abnormalities (Q00–Q99)	77	100.0%	3.0	100.0%	0	0.0%	0.0	0.0%	0	0.0%	0.0	0.0%	77	3.0
		(0.00)				(0.00)				(0.00)				
Symptoms, signs and abnormal clinical and laboratory findings, not elsewhere classified (R00–R99)	2,518	86.0%	45.4	65.2%	407	13.9%	24.2	34.8%	3	0.1%	0.0	0.1%	2,928	69.6
		(0.64)				(0.64)				(0.06)				
Injury, poisoning and certain other consequences of external causes (S00–T98)	11,159	84.5%	100.5	77.1%	1,821	13.8%	14.3	10.9%	225	1.7%	15.6	12.0%	13,205	130.4
		(0.31)				(0.30)				(0.11)				
External causes of morbidity and mortality and other	12,549	86.5%	482.4	72.7%	723	5.0%	110.0	16.6%	1,228	8.5%	71.4	10.8%	14,500	663.8
		(0.28)				(0.18)				(0.23)				
Total	204,110	85.7%	2,139.3	55.4%	18,498	7.8%	382.7	9.9%	15,594	6.5%	1,341.4	34.7%	238,202	3,863.4
		(0.07)				(0.05)				(0.05)				

^a)^Values from the 2008 Korean Health Panel; ^b)^Units: thousand US dollars; ^c)^ICD code: international classification of diseases code.

Table 1 illustrates the cost of medical care by disease group also. The cases of TKM use were varied by disease group. The proportion of TKM use was highest in diseases of the nervous system (G00–G99), with 25.1 ± 0.75% of the total cases with nervous system diseases using outpatient services. The prevalence of TKM use was also high in diseases of the musculoskeletal system and connective tissue (M00–M99) (22.4 ± 0.19%); symptoms, signs and abnormal clinical and laboratory findings not elsewhere classified (R00–R99) (13.9 ± 0.64%), and injury, poisoning and certain other consequences of external causes (S00–T98) (13.8 ± 0.30%). These four disease groups represented three quarters of all TKM outpatient service use. However, none of the cases with certain conditions originating in the perinatal period (P00–P96) and with congenital malformations, deformations and chromosomal abnormalities (Q00–Q99) was determined as TKM use. The cost of TKM outpatient use followed a similar pattern. It varied from 0% for cases with certain conditions originating in the perinatal period (P00–P96) and with congenital malformations, deformations and chromosomal abnormalities (Q00–Q99), to 34.8% for cases in the case of symptoms, signs and abnormal clinical and laboratory findings not elsewhere classified. Generally, the proportion of TKM outpatient service costs was higher than the proportion of TKM use cases, The following disease groups, the cost of TKM exceeds 20% of total cost in each disease group: diseases of the blood and blood-forming organs and certain disorders involving immune mechanisms (D50–D89); diseases of the nervous system; diseases of the musculoskeletal system and connective tissue; and symptoms, signs and abnormal clinical and laboratory findings not elsewhere classified. Among these groups, treating diseases of the musculoskeletal system and connective tissue cost the most at 0.1 million US dollars (Table 1).

Table 2 displays the cases and proportion of cases, and the cost and proportion of cost by medical service category for the top 20 diseases. The top 20 diseases were listed by cases of outpatient visits for TKM use. Among these diseases, the cases of TKM use were highest for soft tissue disorders (M60–M79) (3,798 cases) and other dorsopathies (M40–M49, M53–M54) (3,183 cases). There were also a large number of TKM use cases in arthrosis (M15–M19); dislocations, sprains and strains of specified and multiple body regions (S03, S13, S23, S33, S43, S53, S63, S73, S83, S93, T03); cervical and other intervertebral disc disorders (M50–M51); intracranial hemorrhages (I60–I62); and other diseases of the circulatory system (I85–I99). Of TKM use cases diagnosed with one of the top twenty diseases, the highest proportion of TKM use was shown in the use as the invigorant (98.6 ± 0.51%), followed by cases with nerve, nerve root and plexus disorders (G50–G59) (68.7 ± 1.90%), intracranial hemorrhage (59.3 ± 1.31%) and other diseases of the circulatory system (49.0 ± 1.45%); and soft tissue disorders (33.1 ± 0.44%). The cost of TKM showed a similar pattern, in that the amount and proportion of TKM costs were highest for the use as the invigorant (99.7%); followed by nerve, nerve root and plexus disorders (70.1%); and other diseases of the circulatory system (64.0%) (Table 2).

**Table 2:** **Number of outpatient cases and cost of outpatient medical services for the top 20 diseases treated with Traditional Korean Medicine** ^**a)**^

**Disease Name (ICD code** ^**c)**^ **)**	**Western medicine use**				**Traditional Korean medicine use**				**Dental and other medical service use**				**Total**	
	**Number**	**Proportion of number (S.E.)**	**Cost** ^**b)**^	**Proportion of cost**	**Number**	**Proportion of number (S.E.)**	**Cost** ^**b)**^	**Proportion of cost**	**Number**	**Proportion of number (S.E.)**	**Cost** ^**b)**^	**Proportion of cost**	**Number**	**Cost** ^**b)**^
Migraines and other headache syndromes (G43–G44)	939	80.3% (1.16)	18.8	79.8%	229	19.6% (1.16)	4.8	20.2%	1	0.1% (0.09)	0.01	0.0%	1,169	23.6
Nerve, nerve root and plexus disorders (G50–G59)	181	30.3% (1.88)	2.5	29.7%	410	68.7% (1.90)	5.9	70.1%	6	1.0% (0.41)	0.02	0.2%	597	8.5
Intracranial hemorrhage (I60–I62)	543	38.9% (1.30)	5.9	57.0%	828	59.3% (1.31)	4.0	38.6%	26	1.9% (0.36)	0.45	4.4%	1,397	10.3
Cerebral infarction (I63)	682	82.0% (1.30)	8.6	72.3%	147	17.7% (1.32)	3.3	27.6%	3	0.4% (0.21)	0.01	0.1%	832	11.8
Other diseases of the circulatory system (I85–I99)	596	50.3% (1.45)	5.0	36.0%	581	49.0% (1.45)	8.9	64.0%	8	0.7% (0.24)	0.01	0.1%	1,185	13.9
Other acute upper respiratory infections (J00–J01, J05–J06)	48,393	99.3% (0.04)	150.8	94.9%	300	0.6% (0.04)	7.3	4.6%	39	0.1% (0.01)	0.75	0.5%	48,732	158.8
Other diseases of the nose and nasal sinuses (J30–J31, J33–J34)	4,335	94.1% (0.35)	20.1	66.2%	267	5.8% (0.34)	10.2	33.5%	5	0.1% (0.05)	0.07	0.2%	4,607	30.3
Gastritis and duodenitis (K29)	3,603	96.5% (0.30)	31.3	92.0%	118	3.2% (0.29)	2.7	7.9%	11	0.3% (0.09)	0.04	0.1%	3,732	34.0
Other diseases of the esophagus, stomach and duodenum (K20–K23, K28, K30–K31)	1,813	91.7% (0.62)	19.4	75.9%	158	8.0% (0.61)	5.6	22.0%	6	0.3% (0.12)	0.55	2.1%	1,977	25.6
Other diseases of the skin and subcutaneous tissues (L10–L99)	7,207	95.9% (0.23)	68.5	82.1%	302	4.0% (0.23)	14.9	17.9%	6	0.1% (0.03)	0.03	0.0%	7,515	83.4
Rheumatoid arthritis and other inflammatory polyarthropathies (M05–M14)	1,317	88.2% (0.83)	9.2	84.1%	175	11.7% (0.83)	1.7	15.9%	1	0.1% (0.07)	0.01	0.1%	1,493	10.9
Arthrosis (M15–M19)	11,227	84.0% (0.32)	59.6	77.8%	2,062	15.4% (0.31)	16.8	21.9%	76	0.6% (0.07)	0.23	0.3%	13,365	76.6
Other disorders of the joints (M00–M03, M22–M25)	830	74.2% (1.31)	7.5	70.9%	288	25.7% (1.31)	3.1	29.0%	1	0.1% (0.09)	0.00	0.0%	1,119	10.5
Cervical and other intervertebral disc disorders (M50–M51)	4,359	77.5% (0.56)	60.7	68.1%	1,254	22.3% (0.56)	28.4	31.8%	8	0.1% (0.05)	0.15	0.2%	5,621	89.3
Other dorsopathies (M40–M49, M53–M54)	9,514	74.2% (0.39)	64.3	66.4%	3,183	24.8% (0.38)	32.1	33.2%	127	1.0% (0.09)	0.36	0.4%	12,824	96.8
Soft tissue disorders (M60–M79)	7,664	66.7% (0.44)	52.1	61.6%	3,798	33.1% (0.44)	32.4	38.3%	23	0.2% (0.04)	0.12	0.1%	11,485	84.7
Dislocations, sprains and strains of specified and multiple body regions (S03, S13, S23, S33, S43, S53, S63, S73, S83, S93, T03)	3,328	72.3% (0.66)	29.4	73.1%	1,271	27.6% (0.66)	10.4	25.8%	6	0.1% (0.05)	0.46	1.2%	4,605	40.2
Other injuries of specified, unspecified and multiple body regions (S00–S01, S04, S09–S11, S14–S16, S19–S21, S24–S25, S29–S31, S34–S35, S39–S41, S44–S46, S49–S51, S54–S56, S59–S61, S64–S66, S69–S71, S74–S76,S79–S81, S84–S86, S89–S91, S94–S96,S99, T00–T01, T06–T07, T09, T11, T13–T14)	2,526	90.1% (0.56)	23.4	67.8%	135	4.8% (0.40)	1.3	3.9%	143	5.1% (0.42)	9.77	28.3%	2,804	34.5
Sequelae of injuries, poisoning and other consequences of external causes (T90–T98)	1,810	85.1% (0.77)	13.1	89.1%	310	14.6% (0.76)	1.4	9.7%	8	0.4% (0.13)	0.16	1.1%	2,128	14.7
the use for the invigorant	7	1.3% (0.47)	0.1	0.1%	547	98.6% (0.51)	96.7	99.7%	1	0.2% (0.18)	0.18	0.2%	555	97.0

^a)^Values from the 2008 Korean Health Panel; ^b)^Units: thousand US dollars; ^c)^ICD code: international classification of diseases code.

**Figure 1:**
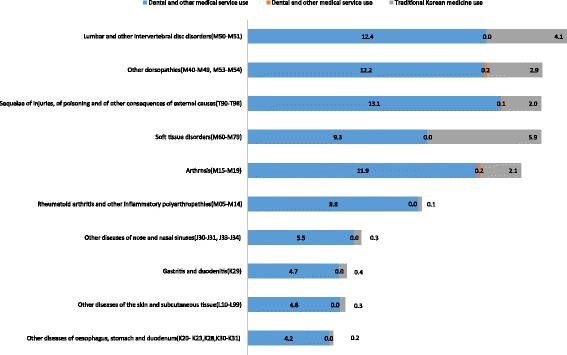
Average number of outpatient visits per year per person for top 10 chronic diseases treated with traditional Korean Medicine.

**Figure 2:**
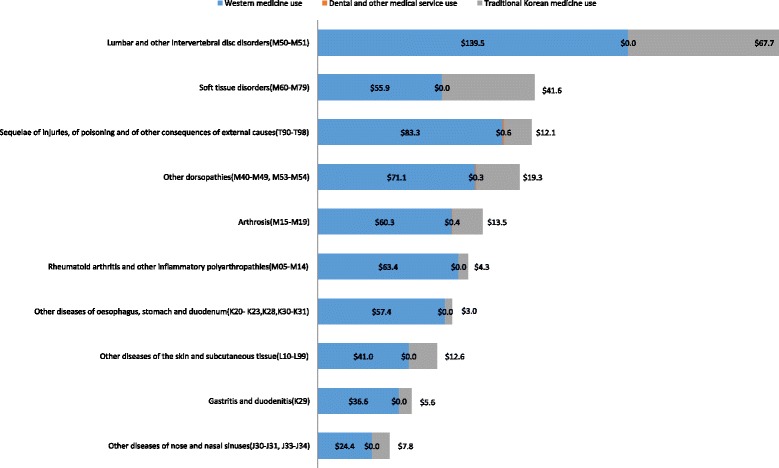
Average cost of outpatient visit per year per person for top 10 chronic diseases treated with traditional Korean Medicine.

The annual number of outpatient cases and the cost of outpatient services of chronic disease patients were identified. The average annual cost and number of cases of the top ten chronic diseases using TKM was high was presented in Figures 1 and 2. Lumbar and other intervertebral disc disorders patients represented the highest annual number of outpatient cases, at 16.5 (±1.87) outpatient visits annually among the diseases selected. Other dorsopathies were the second most common disease (15.2 ± 1.19) visits per year) and sequelae of injuries, poisoning and other consequences of external causes (T90–T98); arthrosis; and soft tissue disorders followed. The frequency of TKM use varied and patients with soft tissue disorders most frequently used TKM, with 5.9 ± 1.00 visits and 38.6% of total visits due to soft tissue disorders. Annual use of TKM was also higher for other dorsopathies and sequelae of injuries, poisoning and other consequences of external causes (Figure 1). In the case of cost, the average annual cost of cervical and other intervertebral disc disorders for outpatient service was highest at 207.2 ± 30.67 per year. The patients with lumbar and other intervertebral disc disorders paid the most for TKM treatment also. They paid $67.7 ± 24.88 per year for TKM treatment, while patients with other dorsopathies paid $19.3 ± 3.18 per year and patients with arthrosis paid $12.1 ± 4.95 per year for TKM treatment (Figure 2).

## Discussion

TKM represented 0.7% of the cases and 1.0% of the admission costs for patients that required admission to a health care institution. For patients requiring emergency services, TKM represented only 0.4% of the cases and 0.1% of the costs. However, in terms of outpatient services, the proportion of TKM use was relatively large.

TKM accounted for 7.8% of outpatient services and 9.9% of costs associated with outpatient services. The following disease groups were associated with frequent users and high costs: diseases of the musculoskeletal system and connective tissue; diseases of the nervous system; and symptoms, signs and abnormal clinical and laboratory findings. In particular, outpatients with soft tissue disorders, other dorsopathies, and arthrosis frequently used TKM. Among patients with chronic diseases, annual TKM use exceeded three visits in cervical and other intervertebral disc disorders, and in soft tissue disorders. Patients with these two types of chronic disease paid more than $30.0 annually for TKM treatment.

Because of the difference in disease classification systems, it can be difficult to determine the relative preference and medical service use patterns of TKM and Western medicine by disease group. In this study the relative preference and medical service use characteristics of TKM and Western medicine were determined using a uniform disease classification system adopted by the Korea Health Panel, showing that the relative proportion of TKM use is highest in the musculoskeletal and nervous system disease groups. The most frequent TKM use cases are those due to musculoskeletal diseases; the musculoskeletal disease group accounted for more than half of TKM use in outpatient services in 2008. These results suggest that the use of TKM or CAM is considered when evaluating treatment options for certain diseases. For example, treatment of the musculoskeletal system was estimated at 6.89 billion dollars in 2008, using claims data that excludes TKM [19]. However, according to this study, TKM represents one third of total musculoskeletal outpatient services. The total economic burden of treating musculoskeletal system diseases increases to 8.1 billion dollars when TKM is taken into account. Understanding the frequency with which certain diseases are treated is critical to create adequate policy intervention, and thus TKM use should be considered when estimating the economic burden of certain diseases. This is relevant not only in Korea, but in other countries where the prevalence of CAM is high.

These results concur with previous findings indicating that musculoskeletal problems are one of most common reasons patients use CAM in Korea [11]. However, in previous studies, CAM was also an important treatment for obstetric/gynecological and endocrine disease groups, while this study found TKM use in these disease groups to be relatively low. This could result from the different types of service included in the study. Previous research included most types of CAM, such as TKM, diet therapies, and herbal medications [11]. This study only included TKM conducted by an DKM.

This study also included patients with chronic diseases, some of whom used TKM for 10–30% of their total medical outpatient services. For example, cervical disc disorder patients paid an average of $64.9 annually for TKM. Patients with soft tissue disorders such as muscle pain also used TKM an average of 4.6 times annually. A study conducted in Singapore found that more than half of those surveyed would hypothetically prefer CAM to Western medicine for back, joint and ankle sprains [21]. Also another study conducted in Taiwan which use claims data showed that main disease group for traditional Chinese medicine were respiratory diseases such as acute sinusitis and musculoskeletal diseases [22]. Also musculoskeletal disease and injury were the most common causes of acupuncture use in Taiwan [23]. Musculoskeletal diseases are the most common causes of CAM use which includes massage, vitamins, acupressure, health food and Kampo in Japan also [24]. In spite of the diversity of research method between studies, the preference for CAM for musculoskeletal diseases is similar in Korea and other countries.

TKM is preferred for outpatient treatment rather than for emergency services or services that require admission to a hospital. More specifically, musculoskeletal diseases such as soft tissue disorders, other dorsopathies, and arthrosis are the most common reasons a patient uses TKM. These diseases include symptoms such as back pain, neuralgia, and general muscle pain. In general, TKM is used to treat diseases that threaten the quality of life rather than diseases that threaten life itself. The characteristics of TKM use in Korea support previous reports on CAM use in Korea and in other countries [12,21,25]. In Korea, the main purpose of CAM is to prevent disease and promote general health [12]. In Singapore, the primary reason for CAM use was maintenance of health rather than treatment of illness. Even only TKM which could be more official part of CAM is only regarded in this study, main reasons of TKM use are generally less life threatening diseases.

Costs associated with TKM were approximately 0.4 million dollars in Korea Health Panel. These costs of Korea Health Panel mean that out-of-pocket costs for TKM use would be 916.1 million dollars for total Korean population in 2008. In addition, when the rate of health insurance coverage at an TKM hospital (41.5%) and clinic (67.7%) were taken into account, then the direct medical cost that Korean paid for TKM use is estimated at 2.6 billion dollars, which is 0.3% of the Korean gross domestic product in 2008 [26,27].

This study used the primary diagnosis as stated by the patient to estimate the disease-specific burden. A similar methodology was used in previous studies and for the official statistics of the National Health Insurance Corporation [19,28]. However, the medical services used by a patient could be affected by comorbidity and not just the primary diagnosis. Furthermore the disease definition by patients could raise the question on the accuracy of diagnosis. And because our study is the cross sectional study, the trend of TKM use could not be estimated. In addition, the use of a medical institution such as a health care center that offers both TKM and Western medicine was regarded as other medical service use, which could have confound the estimates of TKM use. Also, the Korea Health Panel does not include information about payments by the insurer, and therefore total expenditures associated with TKM could not be measured accurately. As well, because we focus on the disease specific differences of TKM use, the characteristics of TKM users, the treatment modalities of TKM and the difference between TKM users and non-users are not analyzed. And the efficacy of TKM or the treatment modality among TKM are not analyzed this study. Therefore, further researches on these subjects are needed.

## Conclusion

Though TKM represents a substantial part of Korean health care, information about disease-specific use of TKM and Western medicine is not well known due to differences in disease classification systems between the two types of medicine. This study used data from the Korea Health Panel and determined that TKM represented only a small proportion of emergency services and services requiring admission to a hospital, though it plays a substantial role in outpatient medical care. The total number of cases and patient expenditures indicate that TKM is used as an important modality for treating musculoskeletal and nervous system diseases, including soft tissue disorders and arthrosis. The characteristics of TKM use in Korea are similar to other countries. TKM use should be considered when estimating the economic burden of certain diseases, particularly in countries with parallel health care systems such as Korea. To the best of the author’s knowledge, this is the first study that determined the actual preference of TKM use in patients with chronic diseases in Korea in the adults.

## References

[1] WHO Health Organization. WHO Traditional Medicine Strategy 2002-2005. Geneva: WHO Health Organization; 2002. [http://www.wpro.who.int/health_technology/book_who_traditional_medicine_strategy_2002_2005.pdf]

[2] What Is Complementary and Alternative Medicine? [https://nccih.nih.gov/health/integrative-health]

[3] Molassiotis A, Fernadez-Ortega P, Pud D, Ozden G, Scott JA, Panteli V (2005). Use of complementary and alternative medicine in cancer patients: a European survey. Ann Oncol.

[4] National Center for Complementary and Alternative Medicine (2011). NCCAM third strategic plan: 2011–2015 exploring the science of complementary and alternative medicine.

[5] Cheung F (2011). TCM: made in China. Nature.

[6] Chang H, Kwon YD, Yoon SS (2011). Use of acupuncture therapy as a supplement to conventional medical treatments for acute ischaemic stroke patients in an academic medical centre in Korea. Complement Ther Med.

[7] Bodeker G (2001). Lessons on integration from the developing world’s experience. BMJ.

[8] Park J (2001). Integrated medicine in the East may differ from that in the West. BMJ.

[9] Index Korea. [http://www.index.go.kr/potal/info/idxKoreaView.do?idx_cd=2772]

[10] National Health Insurance Corporation, Health Insurance Review & Assessment Service (2009). 2008 national health insurance statistical yearbook.

[11] Lee SI, Khang YH, Lee MS, Koo HJ, Kang W, Hong CD (1999). Complementary and alternative medicine use in Korea: prevalence, pattern of use, and out-of-pocket expenditures. Korean J Prev Med.

[12] Ock SM, Choi JY, Cha YS, Lee J, Chun MS, Huh CH (2009). The use of complementary and alternative medicine in a general population in South Korea: results from a national survey in 2006. J Korean Med Sci.

[13] International Classification of Diseases (ICD). [http://www.who.int/classifications/icd/en/]

[14] Oriental medicine diagnosis system Second Edition, KOSIS, Daejeon. 1994. https://kssc.kostat.go.kr:8443/ksscNew_web/index.jsp#.

[15] Lee HJ, Park SB, Kim SJ, Ko SY (2006). System analysis of disease classification of oriental medicine diagnosis and study for improvement method. J Korean Soc Qual Assur Health Care.

[16] The Korean standard classification of diseases and causes of death. [https://kssc.kostat.go.kr:8443/ksscNew_web/kssc/main/main.do?gubun=1##]

[17] Classification of 298 diseases. [http://nhiss.nhis.or.kr/down/pp364-374.hw]

[18] Korea health panel. [http://www.khp.re.kr/english/about_01.jsp]

[19] Oh IH, Yoon SJ, Seo HY, Kim EJ, Kim YA (2011). The economic burden of musculoskeletal disease in Korea: a cross sectional study. BMC Musculoskelet Disord.

[20] Korea Exchange bank. Average exchange rate of Won. [http://fx.keb.co.kr/FER1201C.web?q=AC10021C189E00860FC2C696EAE8C3611A601A20DE0076;Ub9r29zb/rgmwDV79jnsofeso87Xf1WXehEP/L7DzTo749hDiKgrX63PJ3EivmGk;Z1gtyPtYXj1Qz23/2d7gX8NgDhg%3D]

[21] Lim MK, Sadarangani P, Chan HL, Heng JY (2005). Complementary and alternative medicine use in multiracial Singapore. Complement Ther Med.

[22] Chen FP, Chen TJ, Kung YY, Chen YC, Chou LF, Chen FJ (2007). Use frequency of traditional Chinese medicine in Taiwan. BMC Health Serv Res.

[23] Chen FP, Kung YY, Chen TJ, Hwang SJ (2006). Demographics and patterns of acupuncture use in the Chinese population: the Taiwan experience. J Altern Complement Med.

[24] Hori S, Mihaylov I, Vasconcelos JC, McCoubrie M (2008). Patterns of complementary and alternative medicine use amongst outpatients in Tokyo, Japan. BMC Complement Altern Med.

[25] Ernst E (2000). Prevalence of use of complementary/alternative medicine: a systematic review. Bull World Health Organ.

[26] Choi KC, Lee HO, Lee SM (2009). Survey on national health insurance patients’ out-of-pocket expenditure 2008.

[27] Gross domestic production. KOSIS, Daejeon. 2014. http://kosis.kr/statHtml/statHtml.do?orgId=101&tblId=DT_2KAA903&conn_path=I2

[28] Lee YH, Yoon SJ, Kim EJ, Kim YA, Seo HY, Oh IH (2011). Economic burden of asthma in Korea. Allergy Asthma Proc.

